# 

**Published:** 1863-01

**Authors:** 


### INDEX TO VOLUME IV.

Page.

Academy of Medicine, Paris, .......... 285
Action of Medicines in the System. By F. W. Headland, .......... 334
Address, Introductory. By N. S. Davis, M.D., .......... 550
Address Valedictory. By F. Mahla, M.D., .......... 65
Adhesive Plaster, Isinglass, .......... 93
Alcohol as Food, .......... 470
Alcoholic Stimulants as Prophylactics, .......... 472
Amaurosis and Smoking, .......... 272, 576
American Medical Association, .......... 58, 118, 246, 273
Amerman, Geo. K., on Injuries of the Head, .......... 434
Amputation of Femur, &c., &c. By A. Fisher, M.D., .......... 129
Amputation of Leg, Unnecessary. By John Swinburne, M.D., .......... 589
Andrews, E., Record of the Surgery of the Battles near Vicksburg, .......... 12
" New Isinglass Adhesive Plaster, .......... 93
" Injuries of Elbow-Joint, Surgical Clinic, .......... 157
" Improved Methods of Treatment in Joint Diseases, .......... 241
" Report on Surgery to the Ill. State Medical Society, .......... 369
" Improved Methods of Treatment in Joint and Spinal Diseases, .......... 417
" Sulphite of Lime as an Antiseptic, .......... 504
" Improved Methods of Treating Deformities, .......... 564, 634
Angular Curvature of Spine, Mechanical Treatment of, .......... 474
Animal Tissue, Artificial Petrifaction of, .......... 413
Ankle, Dislocation of, &c. By J. S. King, M.D., .......... 315
Army, Death-rate Diminished, .......... 286
Army Medical Society, at Jackson, Tenn., .......... 166
Army Surgeons, .......... 412
Army Surgeons, Tribute to, .......... 122
Arsenic in Sulphuret of Antimony, .......... 653
Artificial Limbs, .......... 61
Auto-Ophthalmoscopy, A New Method of, .......... 603

Ball, Extraction of, .......... 350
Ballet Dresses, Poisonous, .......... 350
Balsam Copaiba, Syrup of, .......... 654
Bandage, New Solid, .......... 103
Bartlett, John, on Cases of Cyanosis, .......... 624
Bedford, G. S., on Principles and Practice of Obstetrics, .......... 115
Belladonna in the Treatment of Epilepsy, .......... 405
Books and Pamphlets, .......... 121
Bowel-Affections of Children, connected with Dentition, .......... 498
Bowman John E., on Medical Chemistry, .......... 334
Bowman, Wm. E., on Soft Chancre, .......... 279

Page.
Braithwaite's Retrospect, ........ 169, 474
Brande, Wm. Thomas, on Chemistry, ........ 270
Brain and Nervous System, Lecture on Certain Diseases of, ........ 516
Breast-Pin, Swallowed by a Child, ........ 349
Bright's Disease, Cure by Milk Diet, ........ 202
Bromide of Ammonium, Therapeutic Properties of, ........ 629
Byford, W. H., on Two Cases of Ovariotomy, ........ 76
" on Singular Case of Erysipelas, ........ 495

Calomel and Tartar Emetic in the Army, ........ 201, 272, 277, 342, 402
Camp Fever, alias Scorbutic Fever. By Dr. McBride, ........ 104
Casein Cement, ........ 655
Catalogue of Medical, Surgical, Dental, and Scientific Books, ........ 402
Cerebro-Spinal Meningitis. By N. S. Davis, M.D., ........ 304
Chancres, Treatment of. By William E. Bowman, ........ 279
Characteristic, ........ 201
Chicago Eye and Ear Infirmary, Statistics of, ........ 345
Chicago Medical Society, ........ 118, 444, 588
Chlorate of Soda, ........ 655
Chemistry. By Wm. Thomas Brande, ........ 270
Childs, Prof. H. H., Resignation of, ........ 206
Cholera.—Subcutaneous Injection of Morphia, ........ 603
Cinchona in Java, ........ 285
Circular from Henry Wing, M.D., ........ 60
Circular to Physicians, ........ 61
Clinical Cases in the Mercy Hospital. By N. S. Davis, M.D., ........ 645
Clinical Lectures on Diseases of Women. By J. Y. Simpson, ........ 113
Cold and Wet a Cause of Camp Disease. By C. A. Hunt, M.D., ........ 226
College Appointments, ........ 120
Colocynth, ........ 349
Communications, ........ 60
Consumption, Treatment of, by the Hypophosphites, ........ 282
Contagion of Secondary Syphilis, ........ 124
Creosote in Hospital Gangrene. By C. R. Parke, M.D., ........ 149
Curara in Hydrophobia, ........ 126
Cyanosis, Cases of. By J. Bartlett, M.D., ........ 624

Davis, N. S., on the Pathology of Dropsy, ........ 95
" on Sulphite of Lime in the Treatment of Erysipelas, ........ 161
" on Cerebro-Spinal Meningitis, ........ 304
" on Diphtheria, ........ 392
" Dentition, and its Connection with Bowel-Affections, ........ 498
" Medical Education, &c., ........ 550
" Clinical Cases in the Mercy Hospital, ........ 645
Death of Dr. Charles Hooker, ........ 204
" Dr. Charles Fishback, ........ 205
Deaths in London in 1862, ........ 286
Dentition, and its Connection with Bowel-Affections, ........ 498
Deformities, Improved Methods of Treatment, ........ 564, 634
Deaf Mute Restored to Hearing by Small-Pox, ........ 151
Diphtheria, ........ 285, 392, 491
Diseases of Horses. By George H. Dadd, V.S., ........ 643
Dropsy, and the Pathological Conditions that give Rise to it, ........ 95
Drowning, Instructions for Restoring Persons, ........ 657

Eczema, Chronic. Lectures on, &c., ........ 347, 451, 501
Elaterium, ........ 350
Elbow-Joint, Injuries of. By E. Andrews, M.D., ........ 157

Page.
Electricity, Muscular, ........ 350, 602
Epilepsy, Treatment with Belladonna, ........ 405
Erysipelas, Treatment of, ........ 161, 491, 495
Esculapian Society, Proceedings of, ........ 266
Essence of Mace, Falsification of, ........ 654
Expectoration, The Action of, ........ 285
Explanation, ........ 201
Eye, Lectures on the Diseases of. By H. D. Noyes, M.D., ........ 318
Eye, Report on the Diseases of. By E. L. Holmes, M.D., ........ 296

Femoral Artery, Ligature of, ........ 89
Fever, Scarlet, Complicated with Rheumatism, &c., ........ 409
Fever, Typhoid. By H. Noble, M.D., ........ 289
Fisher, A., on Operations for Injuries of the Head, ........ 1
" on Amputation of Femur, Sulphite of Lime, &c., ........ 129
" Erysipelas, Diphtheria, and Scarlatina, ........ 491
Fractures and Dislocations, Practical Treatise on. By F. H. Hamilton,... 334

Galvanism Applied to the Vocal Cords, ........ 124
Garibaldi Probe, ........ 413
Gastrotomy. By John O'Reilley, ........ 401
Gelsemium in Convulsions, ........ 438
Gleanings from the French Journals, ........ 653
Globe, Statistics of, ........ 127
Gonorrhœal Ophthalmia, ........ 348, 640

Hæmorrhage, Controlling by Position, ........ 641
Hammond, Surgeon-General, ........ 473
Hatch, Ira, on Bromide of Ammonium, ........ 629
Head, Injuries of. By A. Fisher, M.D., ........ 1, 434
Health of San Fraucisco, ........ 661
Hernia, Congenital Inguinal, Radical Cure of, ........ 570
Hospital Gangrene, Erysipelas and Pyæma, Report on, ........ 644
Hospitality of the Profession in Chicago, ........ 278
Holmes, E. L., on Diseases of the Eye, ........ 296
How they Live in New York, ........ 207
Human Longevity. By W. B. Powell, M.D., ........ 544
Hunt, C. A., on Cold and Wet as Causes of Disease, ........ 226
Hydrophobia, Electricity in, ........ 602
Hygiene, Treatise on, , ........ 530
Hypermanganate of Potossa, ........ 656
Hypophosphites in Consumption, ........ 282

Ice as a Therapeutic Agent in Affections of the Throat. By M. K. Taylor, 651
Illinois, To the Medical Profession of, ........ 412
" State Medical Society, ........ 59, 190, 200, 412
Incompatibility of the Sexes, Physiological, ........ 209
Indications of Progress, ........ 587
Influence of Steam on Lead, ........ 653
Inquests in England for 1862, ........ 660
Insane Poor, ........ 349
Insanity and Intemperance. By Andrew McFarland, M.D., ........ 170
Instruction for Enlisting and Disharging Soldiers. By R. Bartholow, M.D. 586
Intestines, Action of Solar Rays on, ........ 476
Iodine, its Uses, ........ 659
Iron and Quinia, Iodide of, Crystalized, ........ 654

Jackson, Samuel, Resignation of, ........ 124
Joint Diseases, Improved Methods of Treatment, ........ 241, 417
Jones, Amos S., on Ligation of Femoral Artery, ........ 89

Page.
Killed, Reported, ... 64
King, James S., on Compound Fracture of the Ankle, &c., ... 315
King of Prussia, Mental Condition of, ... 391

Lead, Influence of Steam on, ... 653
Lime, Sulphite of, ... 129, 161, 504
Lime, Iodide of, ... 283
Lind University, Medical Department of, ... 116, 336
Liquor Bismuthi, ... 475
Logwood as a Deodorizing Agent, ... 123
London Lancet, ... 170, 474
London, Former and Present Mortality of, ... 349
" Medical Times and Gazette, ... 474
Longevity, Human, ... 544

Mahla, F., Address by, ... 65
Malarious Fever, Subcutaneous Injection of Quinine, ... 583
Materia Medica and Hygiene in University of Buffalo, ... 285
Materia Medica, Synopsis of Lectures on. By J. Carson, M.D., ... 644
McFarland, Andrew, on Minor Mental Maladies, ... 353
Medical and Surgical Journal of the War, ... 476
Medical Association of Southern Central N. Y., Transactions of, ... 272
Medical Chemistry, Hand-Book of, ... 334
Medical Colleges in Chicago, ... 410
Medical Communications of Connecticut Medical Society, ... 399
Medical Jurisprudence, Chair of, ... 595
Medical Secrecy, ... 126
Medical Qualifications, ... 61
Medical Society of McLean Co., ... 123
Medical Societies in Chicago, ... 586
Medical Student's Vade Mecum, ... 334
Mercy Hospital, ... 335
Michigan State University, ... 414
Midwifery, Practical Treatise on. By P. Cazeaux, ... 334
Minnie Ball, Glancing of, ... 126
Minor Mental Maladies, ... 353
Mortality of Philadelphia. By Wilson Jewell, M.D., ... 401

Naval Surgeons, Rank of, ... 286
Nævus, by Tartar Emetic, ... 602
New Books, ... 60
Noble, H., on Typhoid Fever, ... 289

Obituary Record, ... 205
Obstetrics, The Science and Art, ... 271
Ohio State Medical Society, Transactions of, ... 169
Ophthalmic Medicine and Surgery, Treatise on, ... 644
Organic Base without Oxygen, ... 314
Ormsby, O. B., Letter from, ... 236
Ovarian Pregnancy. By R. B. Treat, ... 86
Ovariotomy, Two Cases of. By W. H. Byford, M.D., ... 76
Owen, D. C., on Poisoning by the May Apple, ... 389
Oxygen Gas, ... 350

Paralysis, Infantile, ... 579
Parke, C. R., on Creosote in Hospital Gangrene, ... 149
People's Dental Journal, ... 402
Permanganate of Potash as a Disinfectant, ... 474
Peruvian Physicians, ... 286
Pitcher Plant in Small-Pox, ... 346

Page.
Pharmacopœia of the United States, 585
Podophyllum Peltatum as a Poison, 389
Position in Controlling Hæmorrhage, 641
Powell, W. Byrd, on Physiological Incompatibility of Sexes, 209
" " on Remote Cause of Scrofula, 481
" " on Index to Human Longevity, 544
Prescribing, The Art of. By S. Wickersham, M.D., 139
Prince, David, on Ununited Fractures, 611
Protoxide of Maganese, Sulphate of, 655
Pus in Blood, 333

Report of Board of Health of Philadelphia, 1862, 169
Ridgewood's Disinfecting Powder, 595

Samuels, J. J., on Case of Deaf Mute, 151
Sanitary Condition of the West Division of Chicago, 340
San Francisco, Health of, 661
Sclerotitis and Iritis, 659
Scrofulous Forms of Disease, Remote Cause of, 481
Scrotal Tumor, 414
Skin, Diseases of. By E. Wilson, M.D., 271
Small-Pox, To Prevent Pitting in, 466
Smoking, a Cause of Amaurosis, 272
Stimulants in the Army, 448
Strychnine, Physiological Test for, 125
Sulphate of Morphia, Through the Ear, 207
Surgery of Battles near Vicksburg, 12
Surgery, Principles and Practice of. By H. H. Smith, 168
Surgery, Report on. By E. Andrews, M.D., 369
Syphilis, 348

Taylor, M. K., on Ice as a Therapeutic Agent, 651
Thapsus Verbascum, 603
Tobacco, Use of, 63
Treat, R. B., on Ovarian Pregnancy, &c., 86, 89

Ununited Fractures, Treatment of, 611

Vivisection, 285

Wardner, Horace, Surgeon, Ill. Vols., 125
Washington City, 206
Wickersham, S., on Selection and Prescribing of Remedies, 139
Wing, Henry, on Gelsemium in Convulsions, 438
Witt, W. B., Letter from, 240
Wine of Rennet, 468

### BLISS & SHARP,

### DRUGGISTS AND CHEMISTS,

**144 LAKE STREET, CHICAGO,**

Keep constantly on hand a large assortment of

**PURE DRUGS, FINE CHEMICALS,**

THAYER'S FLUID EXTRACTS & SUGAR-COATED PILLS,

**Keith's Preparations,**

**Tiemann's Celebrated Surgical Instruments,**

Trusses, Elastic Stockings, Bandages, &c.

**Particular Attention Paid to Physicians' Orders.**

THE

### CHICAGO MEDICAL EXAMINER.

N. S. DAVIS, M.D., EDITOR.

A MONTHLY JOURNAL

DEVOTED TO THE

EDUCATIONAL, SCIENTIFIC, AND PRACTICAL INTERESTS

OF THE

MEDICAL PROFESSION.

The EXAMINER will be issued during the first week of each month, commencing with January, 1860. Each number will contain 64 pages of reading matter, the greater part of which will be filled with such contents as will directly aid the practitioner in the daily practical duties of his profession.

To secure this object, fully we shall give, in each number, in addition to ordinary original articles, and selections on practical subjects, a faithful report of many of the more interesting cases presented at the Hospitals and College Cliniques. While aiming, however, to make the EXAMINER eminently practical, we shall not neglect either scientific, social, or educational interests of the profession. It will not be the special organ of any one institution, society, or clique; but its columns will be open for well-written articles from any respectable member of the profession, on all topics legitimately within the domain of medical literature, science and education.

Terms, $2.00 per annum, invariably in advance.

